# Downregulation of *Rab23* inhibits hepatocellular carcinoma by repressing SHH signaling pathway

**DOI:** 10.1002/cnr2.1921

**Published:** 2023-10-26

**Authors:** Si‐Jia Liu, Yu‐Wei Zang, Cui‐Jun Huang, Yun‐Jian Liu

**Affiliations:** ^1^ Department of Anesthesiology The Affiliated Hospital of Jiujiang University Jiujiang China; ^2^ Archives of Jiujiang University Jiujiang China; ^3^ Physical Examination Center The First People's Hospital of Jiujiang City Jiujiang China; ^4^ Department of Hepatobiliary Surgery The Affiliated Hospital of Jiujiang University Jiujiang China

**Keywords:** *GLI‐1*, hedgehog signaling, hepatocellular carcinoma, *Rab23*, *smoothened*

## Abstract

Hepatocellular carcinoma (HCC) is the sixth most common malignant tumors and the third leading cause of cancer‐related death worldwide. As an oncogene, Rab23 has been shown to be significantly related to the growth and migration of hepatocellular carcinoma in both in vitro and in vivo studies, but its underlying mechanism remains obscure. In the present study, we examined the effect of inhibiting *Rab23* expression on the pathological progression of HCC. The correlation between liver *Rab23* gene expression and survival probability in human HCC patients was analyzed using the TCGA database and CPTAC database. *Rab23* knockdown hepatocellular carcinoma cell line was generated through lentiviral transduction, then we established a nude HCC xenograft model by subcutaneously implanting the transfected cells. The analysis of gene and protein expression was carried out using Western blot or RT‐qPCR, respectively. Flow cytometry analysis was used to detect the level of apoptosis. The expression levels of key proteins involved in the Sonic Hedgehog (SHH) signaling pathway were assessed. The results showed that HCC patients with low levels of hepatic *Rab23* mRNA and protein had a better survival tendency than those with higher levels of Rab23. Cell proliferations were reduced and apoptosis levels were increased after Knocking down *Rab23* in HCC cell lines. Furthermore, in vivo studies have demonstrated that suppression of the *Rab23* gene results in decreased tumor size, proliferation rate, and reduced levels of SHH‐related proteins Smoothened and GLI‐1. The above results suggest that Rab23 is involved in the pathological progression of HCC as an important regulator of the SHH signaling pathway, which also provides an important research basis for new therapeutic strategies for HCC.

## 1 BACKGROUND

The incidence of hepatocellular carcinoma (HCC) has increased worldwide, not only in East Asia but also in western Europe and the United States. HCC currently ranked sixth in incidence rate and third in mortality rate among all malignant tumors.^1^ It is estimated by The World Health Organization that over 1 million patients will die from HCC by 2030.^2^ Unfortunately, the current diagnosis rate of HCC is very low, and effective clinical treatments are lacking.^3^ HCC is a complex process involving multiple risk factors, including hepatitis B virus (HBV), Hepatitis C virus (HCV), cirrhosis, and alcohol use, which further promote abnormal activation of specific molecular signaling pathways, leading to disruption of the balance of oncogenes and tumor suppressor genes in cells, respectively.^4^ Currently, various oncogenes such as *TP53*, *CTNNB1*, *AXIN1*, and *ARID1A* have been identified to promote the initiation and progression of HCC by inducing abnormalities in cell proliferation, apoptosis, and signal transduction.^5^, ^6^ In addition, providing a clear understanding of the relationship between these oncogenic genes and HCC, as well as comprehending their essential mechanisms in tumor progression, will contribute to the development of targeted treatments and preventive measures for HCC.

*Rab23* is a conserved member of the Ras‐related small GTPase family, which is crucial for membrane trafficking and protein transport.^7^, ^8^, ^9^ In cancer, *Rab23* has been found to be highly expressed in carcinoma cells and is closely related to tumor cell proliferation, migration and apoptosis as an oncogene with tumor‐promoting activity, which makes it a useful cancer biomarker and potential therapeutic target.^9^, ^10^, ^11^, ^12^, ^13^, ^14^ Furthermore, studies have demonstrated that Rab23 represses hedgehog (Hh) activities Via GLI‐2 and promotes the proteolytic cleavage of GLI‐3 into its cleaved repressor form. In addition, *Rab23* also appeared to regulate Hh pathway activity through Smoothened.^15^ Both *Rab23* gene and SHH pathway have also been implicated in tumor invasion and migration of HCC through their regulation of cell growth.^16^, ^17^ However, the potential mechanisms of *Rab23* in HCC are not yet clear.

In previous studies, we have demonstrated that *Rab23* exists in both GDP‐ and GTP‐bound forms. Disrupting this binding or reducing the level of *Rab23* has a significant impact on the expression and nuclear localization of *Gli1* in human liver cancer cell line Hep3B and HepG2, ultimately leading to suppression of cell growth. Therefore, we conducted this study to investigate the role and in vivo molecular mechanism of *Rab23* silencing in HCC.

## 2 MATERIALS AND METHODS

### 2.1 Datasets collecting and preprocessing

The TCGA_LIHC cohort survival analysis for this study used Gene Expression Profiling Interactive Analysis (GEPIA, http://gepia.cancer-pku.cn/) online analysis, with the data from The Cancer Genome Atlas (TCGA, https://portal.gdc.Cancer.gov/). At the same time, the protein data obtained in this study is downloaded from the The Clinical Proteomic Tumor Analysis Consortium (CPTAC, https://proteomics.cancer.gov/programs/) database.

### 2.2 Survival analysis

First, HCC patients were divided into *Rab23* high group and *Rab23* low group according to the median *Rab23* as a cut‐off value. Load the R package “survival”, then create the Surv object, and use Kaplan–Meier to build an overall survival (OS) analysis model to plot the survival distribution curve between the *Rab23* high group and the *Rab23* low group.

### 2.3 Cell culture

Both the liver cancer cell lines Hep3B and HCCLM3 (iCell Bioscience Inc, Shanghai, China) were used in this study. Hep3B cells were grown in high‐glucose DMEM (Corning, China) medium, while HCCLM3 cells were cultured in RPMI‐1640 (Corning, China) medium. Both types of cells were cultured in a medium supplemented with 10% fetal bovine serum and 1% penicillin–streptomycin at a temperature of 37°C and 5% CO_2_.

### 2.4 Lentivirus transfection

Three specific shRNAs (Table 1) were designed to target the *Rab23* gene, and the lentivirus was purchased from BioSCI Res Company (China). Before transfection, approximately 4 × 10^5^ Hep3B and HCCLM3 cells were plated in a 6‐well plated. The lentivirus particles were added to the cells at a multiplicity of infection (MOI) of 10 overnight. Afterward, the medium was replaced with 2 mL of fresh complete medium and incubated for an additional 48 or 72 h. Stable cell lines were obtained after 48 or 72 h of screening using 2 μg/mL puromycin (BeyoTime, China). Infection efficiency was calculated by observing GFP fluorescence under a fluorescence microscope. Quantitative real‐time PCR or Western blotting was used to measure *Rab23* expression in each stable cells line.

**TABLE 1:** shRNA sequences to target the Rab23 gene.

shRNA	Sequences
*shRab23‐1*	AGGGAATGGAGCAGTTGGAAA
*shRab23‐2*	ACAAACAAAGGACCAAGAA
*shRab23‐3*	CAGAACTAACGCATTCAAGTA

### 2.5 Cell proliferation assay

The Cell Counting Kit‐8 (CCK‐8, Gongsheng, China) was used for cell viability analysis following the manufacturer's instructions. In detail, a total of 10 × 10^3^ cells/well of stable cells were plated onto a 96‐well plate for 8 h and then treated with 10 μL of Cell Counting Kit‐8 (CCK‐8) solution (5 mg/mL) for 4 h. After that, the absorbance is measured at 450 nm in a microplate reader (FLx800, BioTek, Winooski, VT, USA).

### 2.6 Flow cytometric analysis

The apoptosis level was evaluated using an Annexin V‐APC Cell Apoptosis detection kit (BeyoTime, China) on the Guava EasyCyte flow cytometer (Millipore Merck, USA). The cells were dissociated with 0.25% trypsin and washed twice with PBS after transfection. The cell pellets were resuspended in binding buffer and then mixed successively with 200 μL of 1× apoptosis buffer. Annexin V‐APC (5 μL) and PI (5 μL) were added, and the cells were stained for 15 min. Then, the cells were resuspended in 800 μL of 1× apoptosis buffer and the final volume was adjusted to 1 mL. Next, 200 μL of the cell suspension was transferred to a 96‐well plate (with three duplicate wells in each group) and subsequently analyzed using a flow cytometer.

### 2.7 Western blot assay

Total protein was isolated from cultured cells using lysis buffer (50 mM Hepes, pH 7.5, 150 mM NaCl, 10% Glycerol, 1% Triton X‐100, 1.5 mM MgCl2, 1 mM EGTA, 10 mM Sodium Pyrophosphate, 100 mM Sodium Fluoride) with a mixture of protease inhibitors including 1 mM PMSF and 10 μg/mL complete protease inhibitor mixture (Roche). The protein concentration was determined using a bicinchoninic acid protein assay kit (TaKaRa, Japan). The protein samples were run on 10% SDS‐PAGE, transferred to PVDF membranes according to standard procedures.

The membranes were blocked with 5% nonfat milk for 1 h at RT and then incubated overnight at 4°C with primary antibodies, including *Rab23* (11101‐1‐AP, Proteintech, USA), *GLI‐1* (SC‐515751, Santa Cruz, USA), alpha *Smoothened* muscle actin (Ab32575, Abcam, USA) and GAPDH antibody (60004‐1‐lg, Proteintech, USA). Subsequently, the corresponding secondary antibodies were added to the membranes after washed by Tris‐buffered saline/Tween‐20 (TBST). Bands were visualized using the ChemiDocTM MP Imaging System (Bio‐Rad, USA). Densitometric analysis was performed by Image J software.

### 2.8 Quantitative real‐time PCR

Total RNA was extracted from cells using TRIzol reagent (Invitrogen, USA), and cDNA was synthesized by Hiscript III All‐in‐one RT SuperMix Perfect for qPCR (Vazyme, China) according to the manufacturers' instruction. The specific primers used are listed as follows: *Rab23* F1 (5′‐GCTTGTGTGCTCGTGTTCTCTA‐3′) and R1 (5′‐CCACTTCGGCTACTACTTTCTCTC‐3′); *GAPDH* F1 (5′‐CCTGGAGAAACCTGCCAAGTA‐3′) and R1 (5′‐TCATACCAGGAAATGAGCTTGAC‐3′). Quantitative real‐time PCR (qRT‐PCR) was preformed to quantify the RNA of interest using the 2^−ΔΔCT^ method and SYBR Green PCR reagents (Vazyme, China).

### 2.9 In vitro studies

#### 2.9.1 Human HCC xenograft model

Eight‐week‐old BALB/c nude mice (GemPharmatech, China) were used for in vivo experiments and divided into two groups (*shCtrl* group and *shRab23* group). The mouse had free access to standard feed and water in a temperature‐controlled facility, which maintained a 12‐hour light and 12‐hour dark cycle. Tumorigenesis was induced by subcutaneous injection of mice with 4 × 10^6^ transfected HCCLM3 cells. The size of the tumor is regularly measured using a caliper at the same time point each week. Optical imaging was captured via the IVIS spectral imaging system (PerkinElmer, USA), and image analysis was conducted using the Living Image version 3.2 software. The nude mice were euthanized by injection of 3.5% aqueous solution of carbachol (0.1 mL/g) when the tumors reached an appropriate size. The tumor size is measured to further calculate the tumor volume using the formula length × width × 0.5. Each tumor tissue will be collected and weighed. All animal experiments were carried out in accordance with the U.K. Animals (Scientific Procedures) Act, 1986 and associated guidelines, EU Directive 2010/63/EU for animal experiments, or the National Institutes of Health guide for the care and use of Laboratory animals (NIH Publications No. 8023, revised 1978).

### 2.10 Histological staining

Collected tumor tissues were paraffin embedded after fixed with 10% formalin fixation, and cut into appropriate thin slices. Hematoxylin and eosin (H&E) and Ki67 staining were performed according to a standardized procedure. Immunohistochemical analysis was used to detect the expression of *Rab23* in tumor tissues using *Rab23* antibody (Proteintech, USA).

### 2.11 Statistical analysis

Descriptive data were presented as means ± standard deviations (SD)s. The professional statistical analyses were performed using GraphPad Prism software version 5.01. Student *t* tests, one‐way or two‐way analysis of variance (ANOVA) were used to analyze the differences between the groups, respectively. A *p* value of <0.05 is considered statistically significant.

## 3 RESULTS

### 3.1 Correlation of hepatic *Rab23* gene expression with survival probability in human liver cancer patients

**FIGURE 1:**
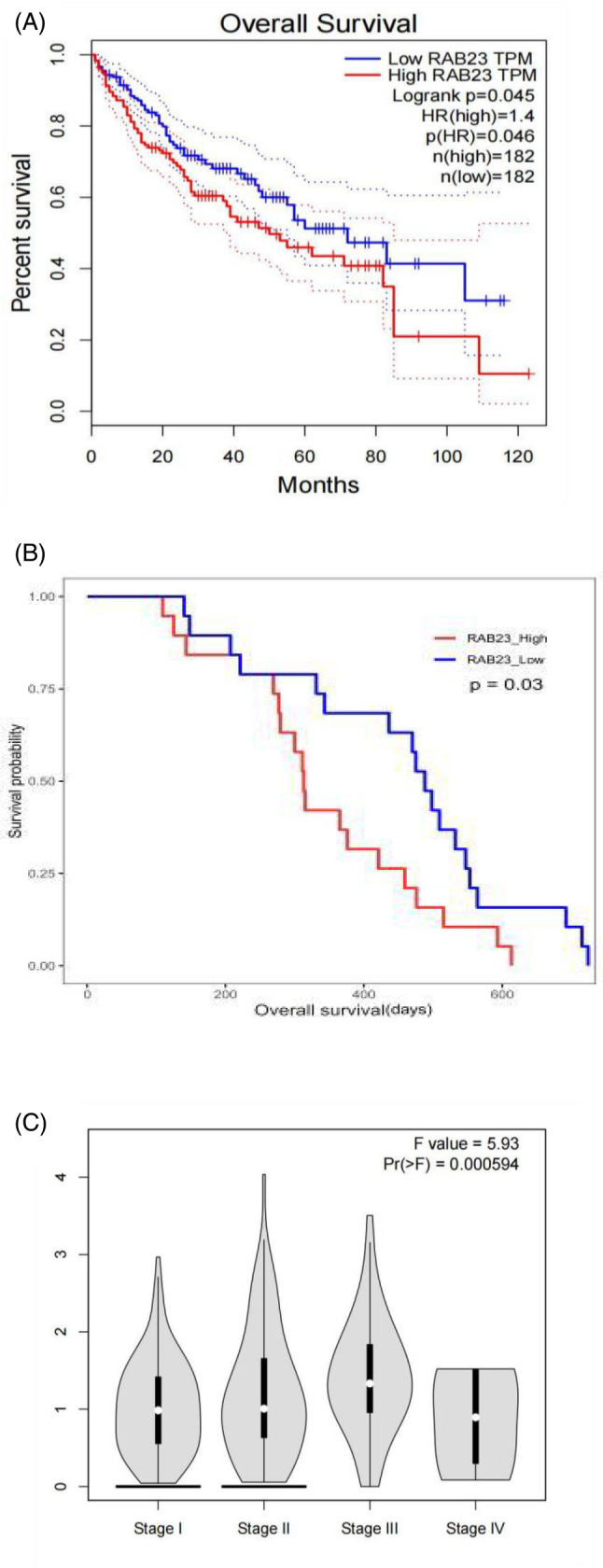
Correlation of hepatic *Rab23* gene expression with survival probability in human liver cancer patients. (A) Kaplan–Meier survival curves comparing high and low *Rab23* RNA expression levels in Gene Expression Profiling Interactive Analysis. (B) Kaplan–Meier survival curves comparing high and low expression of *Rab23* protein expression levels in The Clinical Proteomic Tumor Analysis Consortium. Patients with lower hepatic *Rab23* mRNA and protein levels had a tendency of better survival than those with higher *Rab23* levels and the p value reached a statistical significance. (C) Differences of *Rab23* RNA expression levels in tumor samples at different stages of hepatocellular carcinoma (HCC). With the stage of the patients becoming higher from 1 to 4, the expression level of *Rab23* is high from low.

As the role of *Rab23* in HCC is not well‐understood, we conducted an analysis to examine the correlation between hepatic *Rab23* gene expression and survival probability in human HCC patients using the TCGA dataset and CPTAC dataset. Our analysis revealed that patients with lower levels of hepatic *Rab23* mRNA and protein had better survival rates compared to those with higher levels of *Rab23* (*p* < 0.05) (Figure 1A,B). Moreover, the expression level of *Rab23* was also shown to be significantly and positively correlated with the stage of HCC (Figure 1C).

### 3.2 Expression of *Rab23* in liver cancer cell lines

**FIGURE 2:**
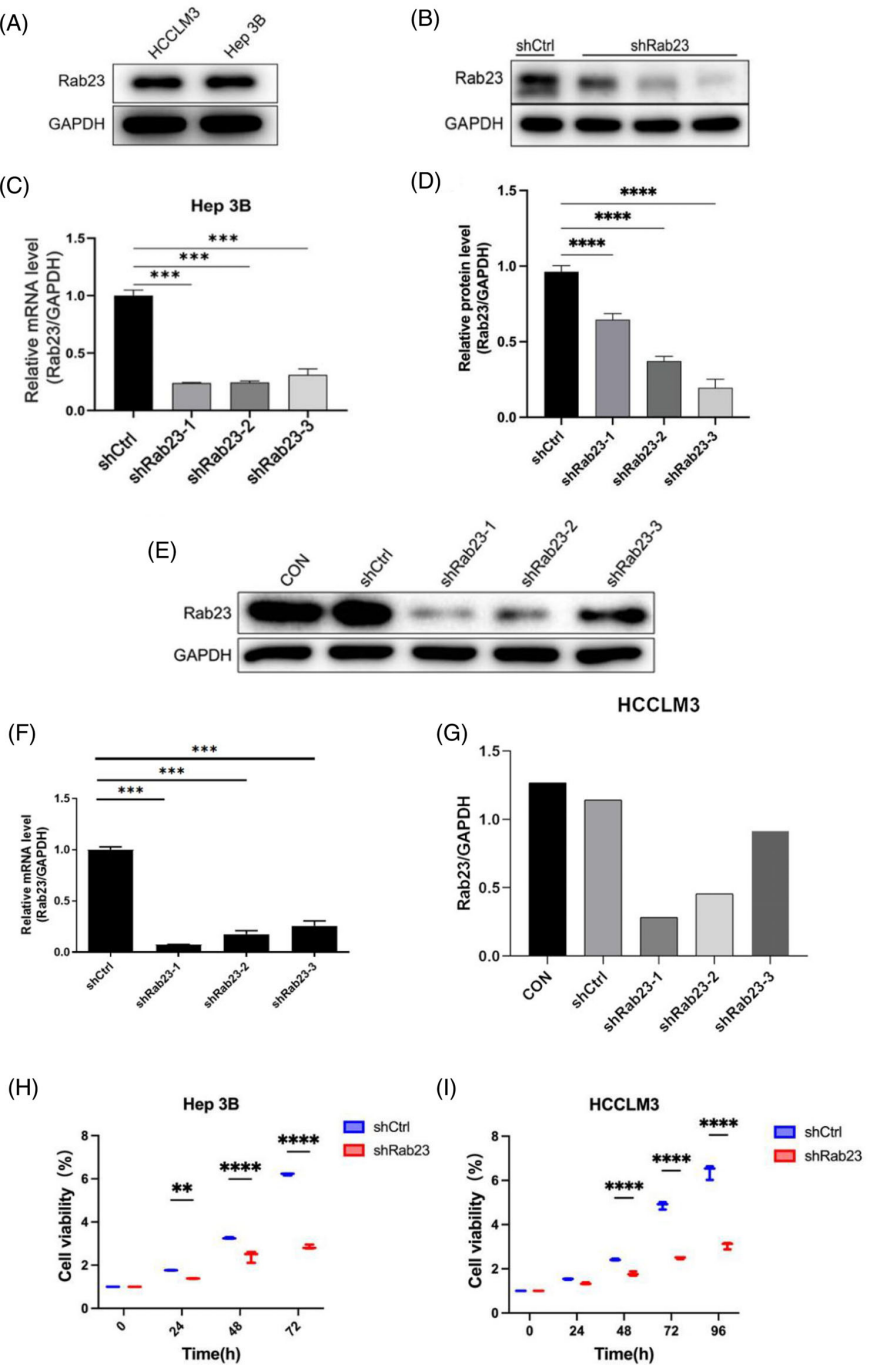
Expression levels of *Rab23* and *shRab23* in HCCLM3 cell lines and Hep3B cell lines, and cell viability analysis of these two cell lines. (A) The protein expression of *Rab23* in HCCLM3 cell lines and Hep3B cell lines was detected by Western blot. (B) The protein expression of *Rab23* in knockdown Hep3B cells was detected by Western blot. (C) The expression of *Rab23* in lentiviral‐transducted Hep3B cells was then measured by qRT‐PCR. (D) Quantification of the expression of *Rab23* protein in transfected Hep3B cells. (E) Western blot of *Rab23* expression in knockdown HCCLM3 cells. (F) The relative mRNA expression of *Rab23* was normalized to that of GAPDH in knockdown HCCLM3 cells. (G) Quantification of the expression of *Rab23* protein in transfected HCCLM3 cells. Cell viability for infected (H) Hep3B and (I) HCCLM3 cell assayed by CCK‐8. The data are presented as the mean ± SD. Significant differences between the shCtrl group and the *shRab23* group: *n* = 3 per group, ***p < 0.001.

We first examined the expression of *Rab23* protein in wild and knockdown cell lines by Western blot analysis, respectively. The results showed that both Hep3B and HCCLM3 cells had similar levels of endogenous *Rab23* protein expression (Figure 2A). We generated *Rab23* knockdown cells in Hep3B cell lines using three different shRNA sequences targeting *Rab23* (*shRab23‐1, shRab23‐2* and *shRab23‐3*). The gene expression of *Rab23* significantly decreased in these knockdown cell lines compared to shCtrl (*p* < 0.001), and the knockdown efficiency is a maximally of 70% (Figure 2C). Moreover, all three shRNAs reduced the protein levels of *Rab23* in cells with different knockdown efficiencies (22% for *shRab23‐1*, 61% for *shRab23‐2*, and 80% for *shRab23‐3*) (Figure 2B,D). We established a *Rab23* knockdown cell line in HCCLM3 cells using the aforementioned shRNA sequences. Compared to the control group, both the gene level (Figure 2F) and protein level (Figure 2E,G) of *Rab23* were significantly decreased. Among them, the knockdown efficiency of *shRab23‐1* was the highest, reaching nearly 90%.

### 3.3 *Rab23* knockdown decreases cell proliferation and induces cell apoptosis in liver cancer cells

**FIGURE 3:**
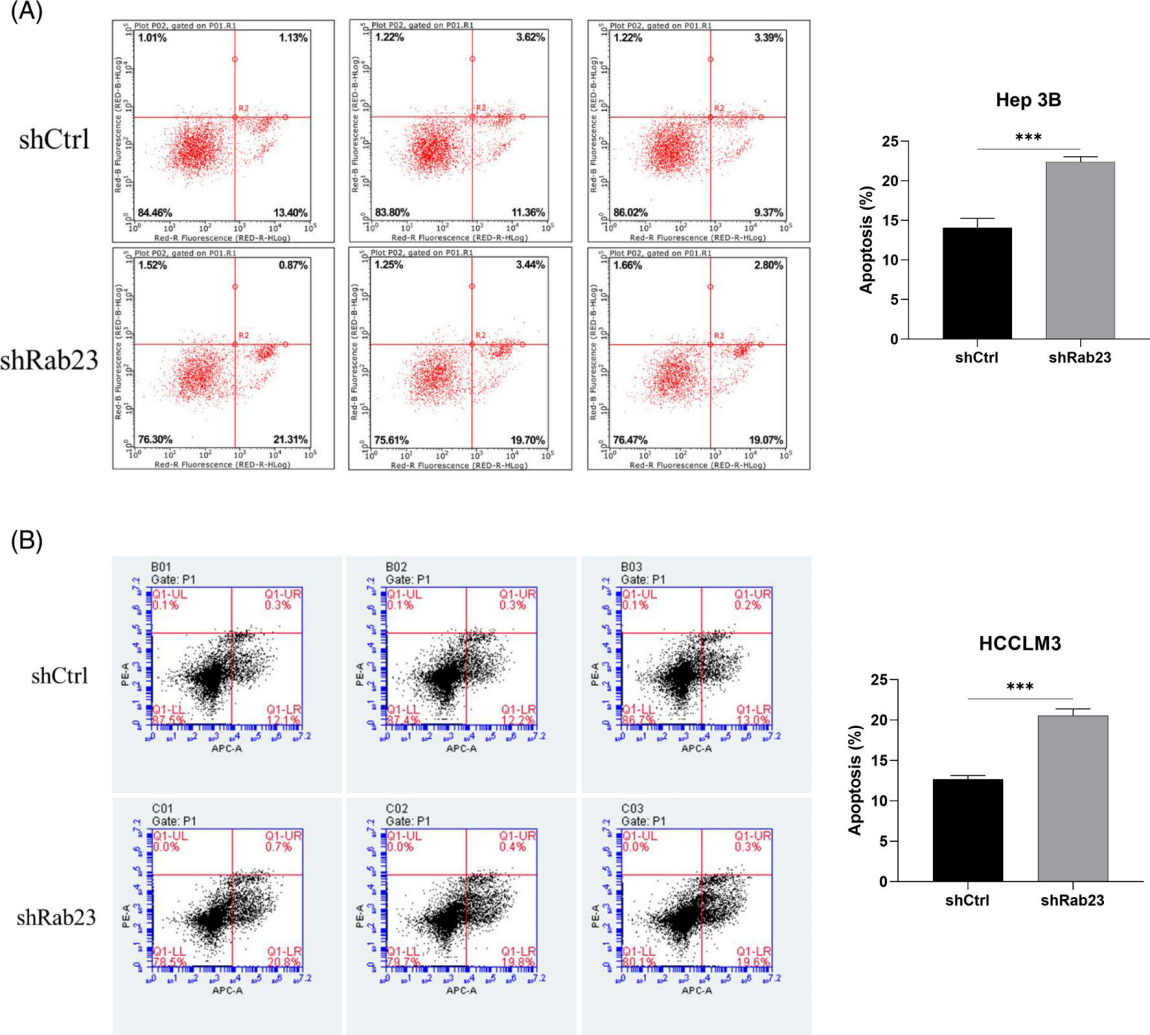
Hep3B cells and HCCLM3 cells were treated with *shRab23*, viability assessment was detected by flow cytometer. Three technical repetitions for each analysis.

We next examined cell proliferation in these *Rab23* knockdown cell lines. The cell proliferation levels of Hep3B cells at 72 h and HCCLM3 cells at 96 h were significantly lower than those of the shCtrl group (*p* < 0.001) (Figure 2H,I). Furthermore, we investigated the impact of *Rab23* knockdown on apoptosis levels in hepatocellular carcinoma cell lines using flow cytometry. The results showed that the apoptosis rate within both Hep3B and HCCLM3 cells was significantly increased in the *shRab23* knockdown group compared to the shCtrl group (*p* < 0.001) (Figure 3A,B).

### 3.4 *Rab23* knockdown suppresses *GLI‐1* expression

**FIGURE 4:**
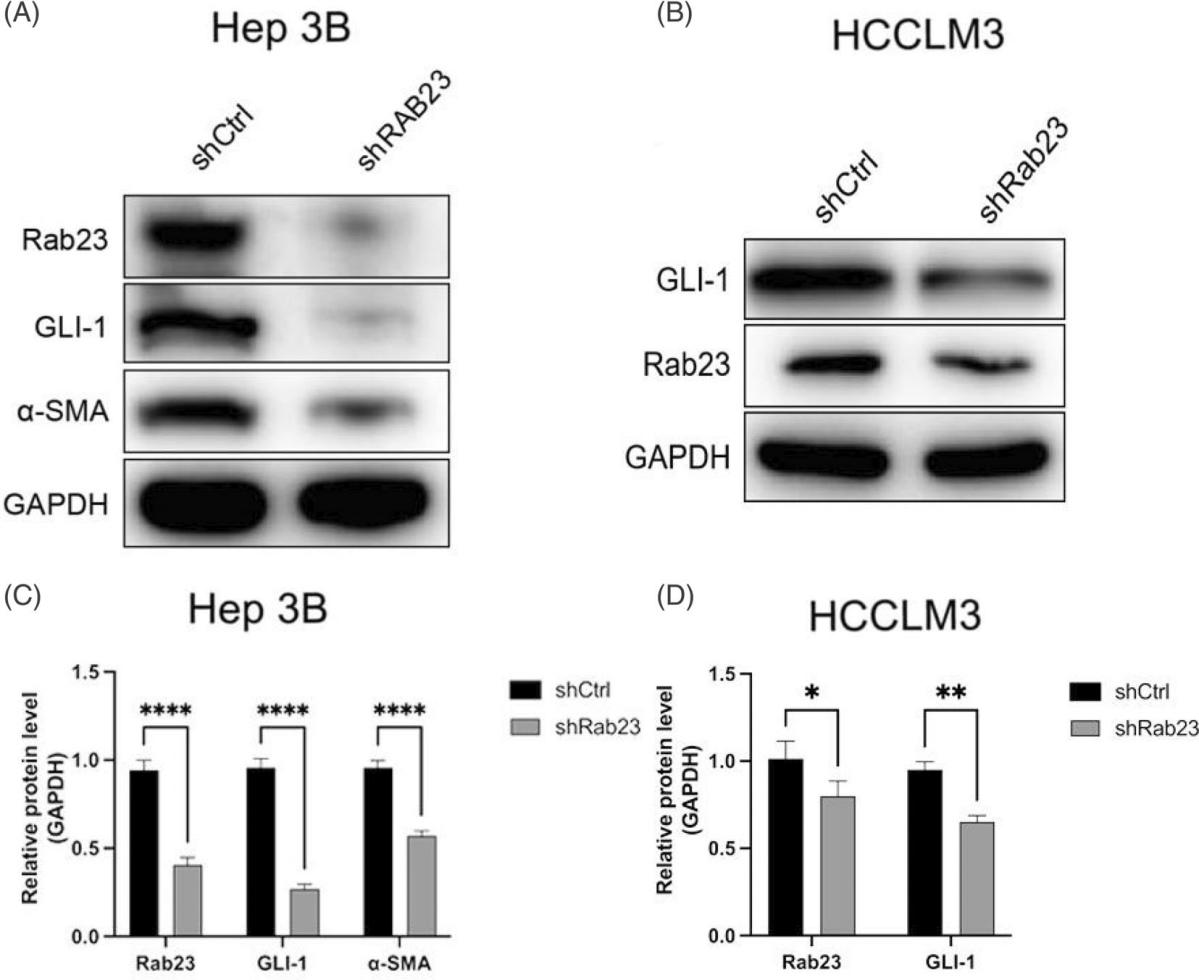
Hep3B cells and were transfected by *shRab23*, protein expression of *Rab23*, *GLI‐1* and *α‐SMA* were detected by Western blot (A). HCCLM3 cells were transfected by *shRab23*, protein expression of *GLI‐1* and *Rab23* were detected by Western blot (B). The average expression levels of *Rab23*, *GLI‐1* and *α‐SMA* in Hep3B cell lines were calculated by the ratio of *Rab23*, *GLI‐1* and *α‐SMA* to GAPDH respectively (C). The average expression levels of *Rab23*, *GLI‐1* in HCCLM3 cell lines were calculated by the ratio of *Rab23*, *GLI‐1* to GAPDH respectively (D). Significant differences between the shCtrl group and the *shRab23* group: *n* = 3 per group, *p < 0.05; **p < 0.01; ***p < 0.001.

To evaluate the effect of *Rab23* knockdown on SHH signaling pathway, the expressions of *GLI‐1* and cancer‐associated fibroblast markers *α‐SMA* in the *shRab23* group were further detected. We found that when compared to WT control, both *Rab23, GLI‐1* and *α‐SMA* protein levels were all significantly downregulated in *shRab23* cells l (*p* < 0.001 in Hep3B cells; *p* < 0.05 (*GLI‐1*) or *p* < 0.01 (*Rab23*) in HCCLM3 cells) (Figure 4A–D).

### 3.5 Proliferation inhibition of BALB/c xenograft tumor with *shRab23* in vivo

**FIGURE 5:**
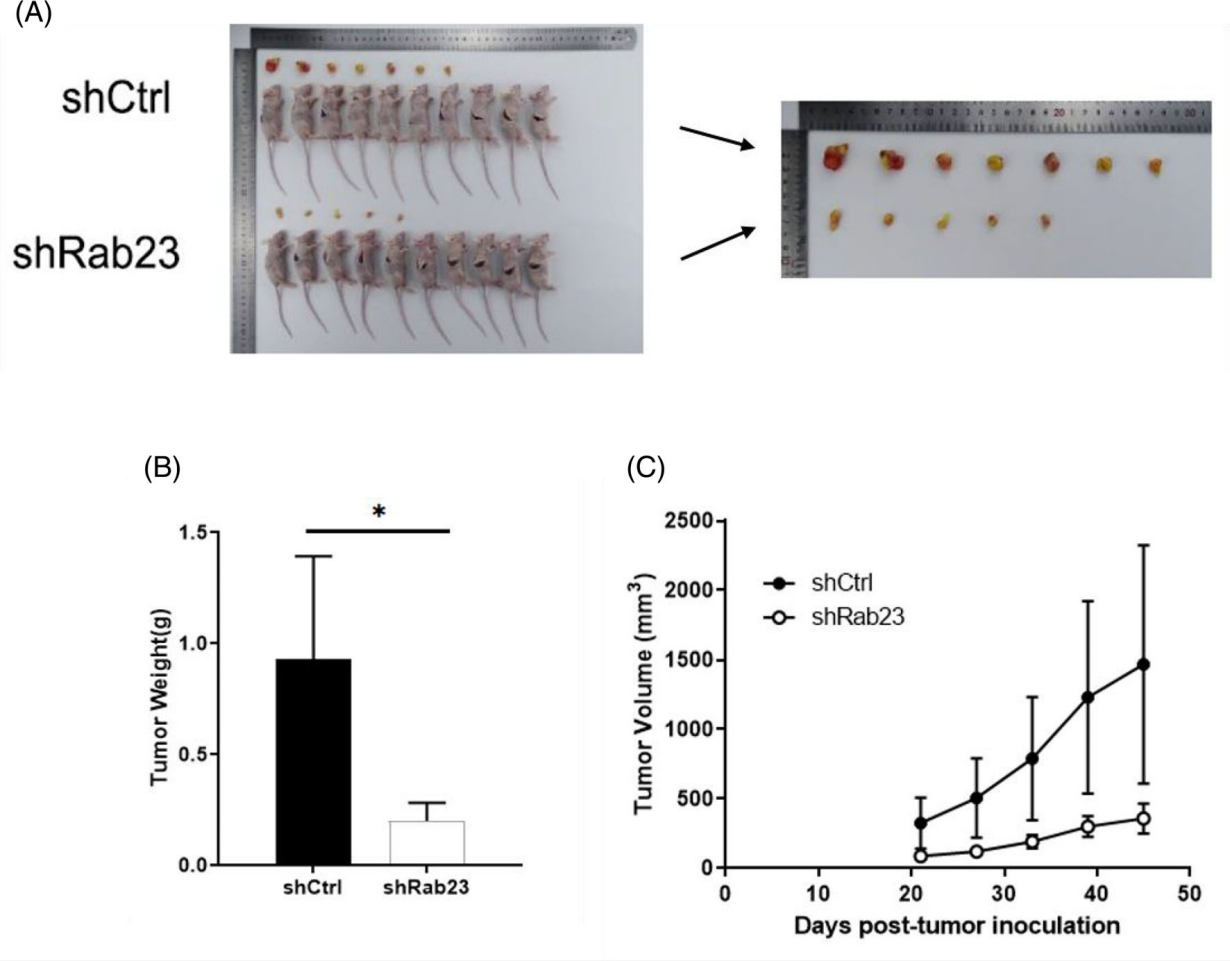
Silencing of *Rab23* inhibited the tumor growth of hepatocellular carcinoma (HCC). Images of mice and tumors from the mice model and tumor size measurement (A). Images of tumor's weight (B). Images of tumor's growth curve (C).

**FIGURE 6:**
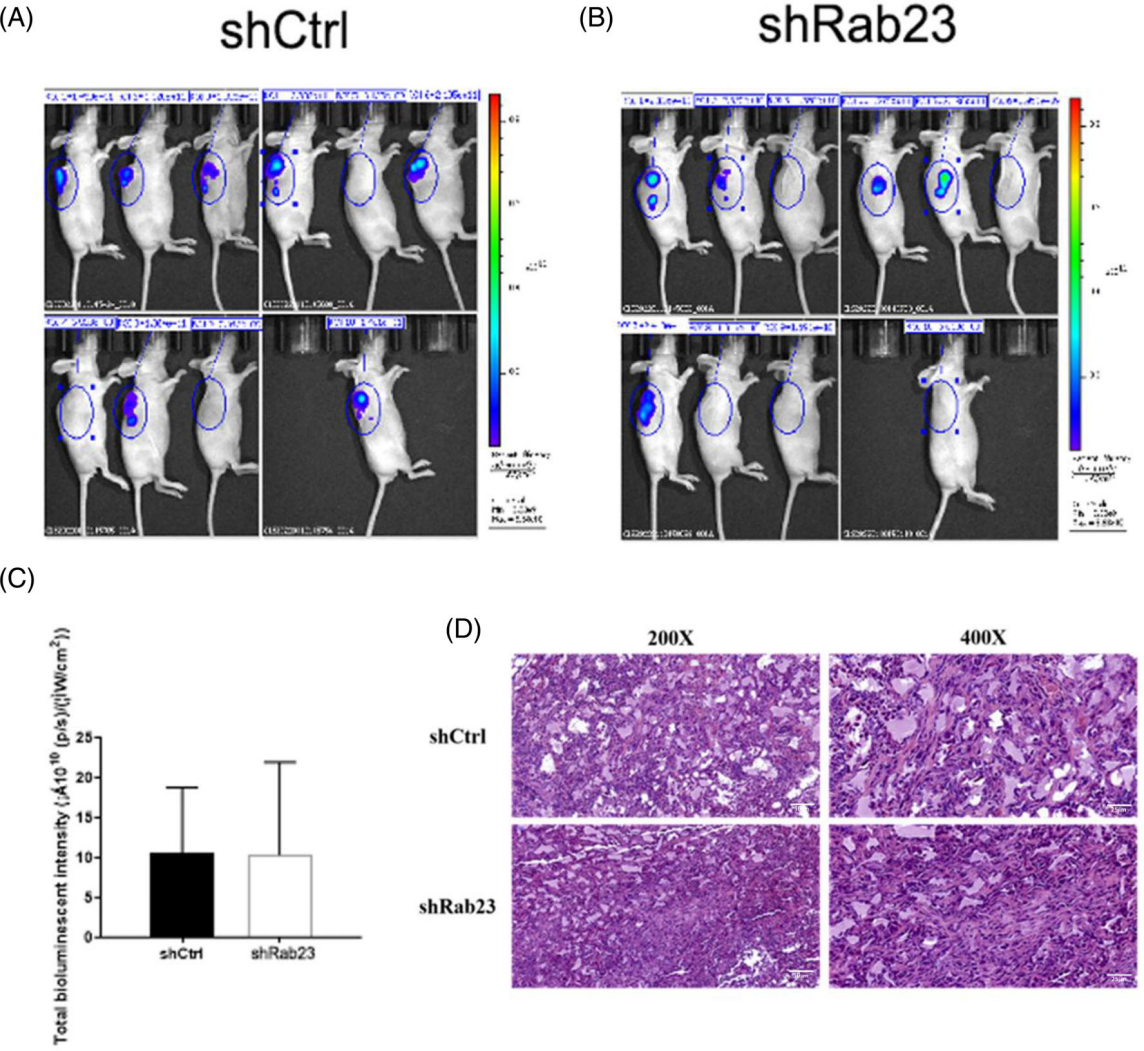
Representative images of fluorescence signals in nude mice detected by the International Veterinary Information Service system. The fluorescence signal intensity shows xenograft tumor size. (A) Tumor model of transplantation with control cells. (B) Tumor model of transplantation with *shRab23* stably cells. (C) Measurement of fluorescence intensity. (D) H&E‐stained liver sections exhibit the hepatic pathology with or without *shRab23‐1* of *Rab23*.

To further validate the suppressive influence of Rab23 on tumorigenesis in vivo, we implanted *Rab23* knockout HCCLM3 cells into nude mice and examined the enlargement of tumor cells. Compared with the shCrtl group, we found that tumors from *shRab23* knockdown cell lines grew more slowly characterized by lower tumor tissue weight (*p* < 0.05) and smaller volume (Figure 5A–C). H&E staining also indicated that tumor cells in the *Rab23* knockout group had significantly reduced irregularly shaped tumor cell nucleosomes, incomplete cytoplasmic membranes, and heterogeneous chromatin structures compared to the control group (Figure 6D). However, according to photon flux detection, there were no significant differences of the metastasis of HCC between two groups (Figure 6A–C).

### 3.6 Silencing of *Rab23* suppress the progression of HCC in vivo via inhibiting the SHH signaling pathway

**FIGURE 7:**
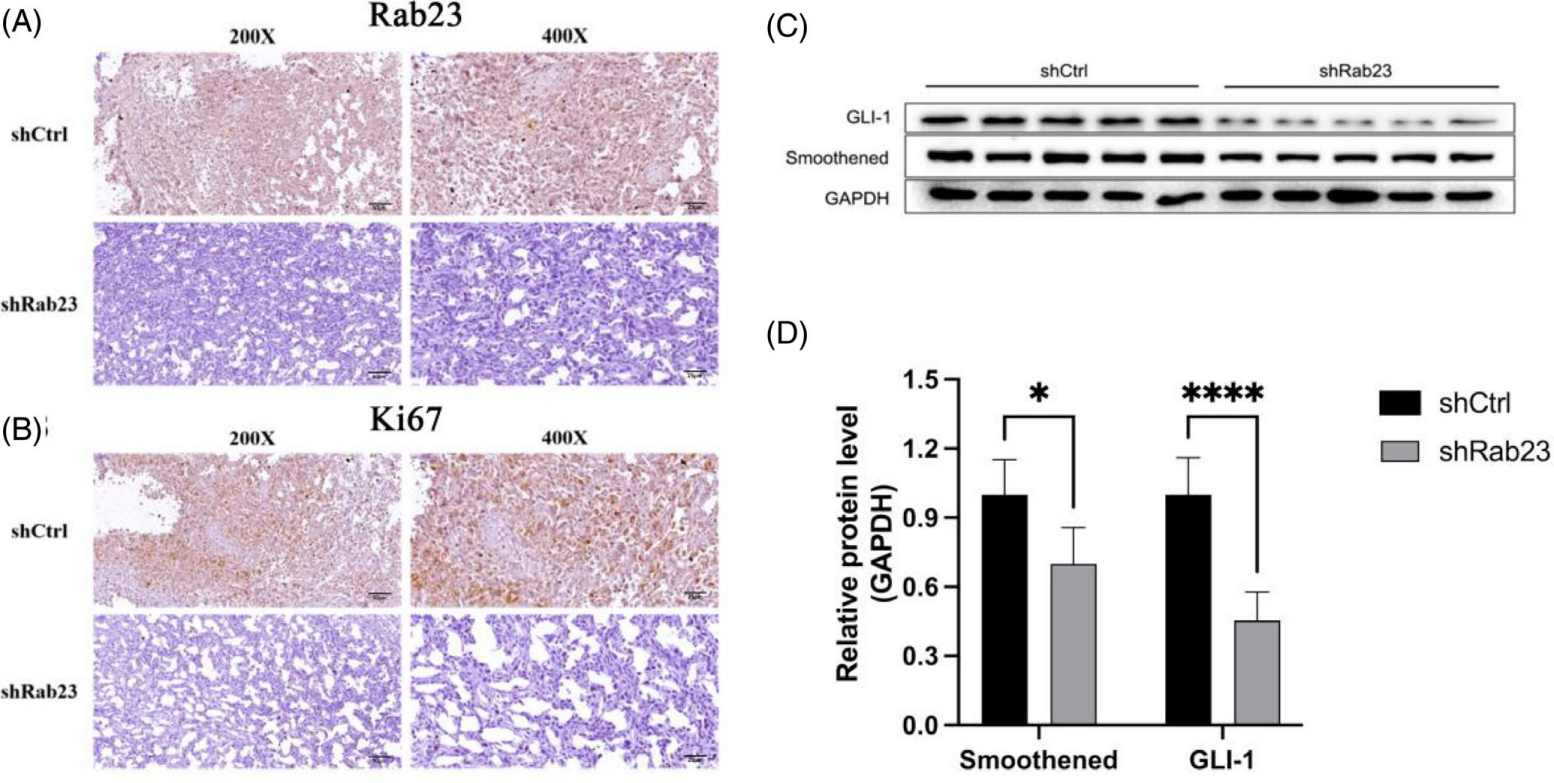
The Sonic Hedgehog (SHH) signaling pathway were suppressed by Downregulation of *Rab23*. (A) The *Rab23* protein expressions in liver cancer tissue with or without *siRab23* were measured by immunohistochemical (IHC) staining. (B) The proliferation index Ki67 protein expressions in liver cancer tissue with or without *siRab23* were detected by IHC staining. (C) *GLI‐1* and *Smoothened* protein expression in liver cancer tissue with or without *siRab23* were checked by western blot; *n* = 5 per group. (D) The average expression levels of *GLI‐1* and *Smoothened* protein in liver cancer tissue were calculated by the ratio of *GLI‐1* and *Smoothened* to GAPDH respectively; *n* = 5 per group.

Immunohistochemical (IHC) staining showed reduced *Rab23* expression in knockout liver cancer cells (Figure 7A), along with decreased levels of KI‐67 (Figure 7B), a proliferation marker for human tumors that has been strongly linked to advanced HCC.^18^ Furthermore, the expression levels of the major SHH pathway proteins *Smoothened* (*SMO*) and *GLI‐1* were also significantly reduced in *shRab23* liver cancer cells compared to the control (Figure 7C,D). Therefore, these findings indicated that Silencing of *Rab23* may suppress the progression of HCC via inhibiting the SHH signaling pathway in vivo.

## 4 DISCUSSION

HCC is a prevalent malignant tumor worldwide and is the primary cause of cancer‐related deaths. In Asia such as China, 5‐year survival rates are reported to be as low as 12%.^19^ Conventional treatments, including resection and anti‐tumor medication, are not yet optimal due to the high diagnosis rate at advanced stages, leading to insignificant improvements in survival rates.

*Rab23* is a small GTPase belonging to the Rab superfamily, whose gene is located on human chromosome 6p12.1.^8^ Over the past few years, it has been confirmed by several studies that *Rab23* is an oncogene that plays a critical role in promoting cancer development.^10^ Furthermore, it has been found to be involved in promoting invasion of various types of cancer including breast cancer, gastric cancer, lung cancer, and so forth.^20^, ^21^, ^22^ Sicklick's study revealed *Rab23* had elevated levels in human liver cancer tissues or liver cancer cells and was closely associated with tumor size.^23^ Zhang et al.^11^ found that overexpression of *Rab23* promoted the migration of Hep3B cells, and this process could be reversed by silencing *Rab23*. Currently, only a limited number of papers have reported on the role of *Rab23* in HCC, and the specific molecular mechanisms by which *Rab23* promotes the progression of liver cancer are far from being elucidated.

In previous study, we found that exhibited a positive expression rate of 53.5% in patients with HCC and was significantly associated with tumor size.^14^ The downregulation of *Rab23* mRNA in Hep3B cells has also been shown to promote apoptosis and inhibit cell growth.^14^ Furthermore, we also demonstrated that the nuclear localization of *Rab23* depends on its GDP/GTP binding state, and that the biological function of *Rab23* is to control GTP hydrolysis as a molecular switch.^16^ In addition, *Rab23* exhibited inhibitory effects on the transcriptional activity of *Gli1* in HepG2 cells, although it was not directly regulated by the SHH signaling pathway.^16^ However, the above studies were all performed in vitro, and there is still a lack of evidence to support the function of *Rab23* in vivo. Therefore, we conducted this present study to gain a deeper understanding of the molecular mechanisms underlying *Rab23* by utilizing Xenograft mouse models.

In our research, we generated a nude mouse xenograft model of HCC by subcutaneously implanting a HCCLM3 cell line with *Rab23* knockdown. The findings demonstrated that *Rab23* knockdown suppressed the growth of cancer cells and increased apoptosis in vivo. Importantly, we found that the deficiency of *Rab23* resulted in a significant decrease in protein levels of both *SMO* and *GLI‐1*. This suggests that *Rab23* plays an essential role in liver cancer development and the SHH signaling pathway, which is consistent with our previous studies.

The SHH pathway is a well‐known signal transduction pathway comprising of main components such as hedgehog (*Hh*), the patched receptor(*Ptc*), a 12‐domain transmembrane receptor, the *Smoothened* receptor(*SMO*), a 7‐domain transmembrane receptor coupled to G protein‐coupled receptor(GPCR), zinc finger transcription factor cubitusinterruptus (Ci), and other components that regulate embryonic development.^24^ Numerous studies have indicated a close correlation between the aberrant activation of the SHH signaling pathway and the occurrence of various types of tumors such as cancers of skin, brain, liver, gallbladder, pancreas, stomach, colon, breast, lung, prostate, and hematological malignancies.^20^, ^21^, ^22^ Sicklick et al. found the SHH signaling pathway plays an important role in the pathogenesis of HCC because of high expression levels of *Ptc*, *SMO*, and *GLI‐1* in liver cancer.^23^ *SMO* is a major component involved in SHH signaling transduction, which can be coupled with G protein‐coupled receptors (GPCRs) to inhibit the activity of suppressor of fused protein (SUFU) and GLI family of transcription factors. When the SHH signaling pathway is activated, *PTCH‐1*, which destabilizes *SMO* and inhibits its activity, is degraded, leading to the activation of G‐protein coupled receptor (GPCR)/*SMO* activity.^25^ This promotes the dissociation of *GLI* from its inhibitor protein SUFU, resulting in the release and transport of *GLI* into the nucleus, triggering downstream target gene expression and activating cellular functions.^26^ However, our results revealed a significant decrease in *SMO* and *GLI‐1* protein levels in tumor tissues, suggesting that *Rab23* plays a negative regulatory role in the SHH signaling pathway and can suppress tumor growth in HCC.

Overall, our study indicate that reducing *Rab23* gene expression inhibit tumor cell growth and promoted apoptosis in vivo by downregulating the SHH signaling pathway. These experimental results present new evidence for the mechanisms of *Rab23* on HCC, which will help to improve the development of therapeutic strategies for patients with hepatocellular carcinoma.

## AUTHOR CONTRIBUTIONS

Yun‐Jian Liu conceived and designed the present study. Si‐Jia Liu analyzed and interpreted data. Yu‐Wei Zang and Cui‐Jun Huang performed experiments, wrote the manuscript. All authors approved the final version to be submitted.

## FUNDING INFORMATION

This study was supported by the Key Science Foundation of Jiangxi Province Department of Education, China (Grant No. GJJ161064) and Natural Science Foundation of Jiangxi Province (No. 20202BABL206092).

## CONFLICT OF INTEREST STATEMENT

The authors have stated explicitly that there are no conflicts of interest in connection with this article.

## ETHICS STATEMENT

All procedures performed in studies involving animal experiments were accordance with the ethical standards of the Research Ethics Committee of the affiliated hospital of Jiujiang University (jjuhmer‐b‐20220503) and with the 1964 Helsinki declaration and its later amendments.

## References

[1] Organization IAfRoCWH . Liver Source: Globocan [EB/OL]. 2020. Accessed October 23, 2023. https://gco.iarc.fr/today/data/factsheets/cancers/11-Liver-fact-sheet.pdf

[2] Sleeman KE , Gomes B , de Brito M , Shamieh O , Harding R . The burden of serious health‐related suffering among cancer decedents: Global projections study to 2060. J Palliat Med. 2021;35:231‐235. 10.1177/0269216320957561 PMC7797611 32945226

[3] Bruix J , Sherman M . Management of hepatocellular carcinoma: an update. Hepatology. 2011;53:1020‐1022. doi:10.1002/hep.24199 21374666 PMC3084991

[4] Alqahtani A , Khan Z , Alloghbi A , Said Ahmed TS , Ashraf M , Hammouda DM . Hepatocellular carcinoma: molecular mechanisms and targeted therapies. Medicina. 2019;55:55. 31450841 10.3390/medicina55090526 PMC6780754

[5] Khemlina G , Ikeda S , Kurzrock R . The biology of hepatocellular carcinoma: implications for genomic and immune therapies. Mol Cancer. 2017;16:149. 28854942 10.1186/s12943-017-0712-x PMC5577674

[6] Shibata T . Genomic landscape of hepatocarcinogenesis. J Hum Genet. 2021;66:845‐851. 33958712 10.1038/s10038-021-00928-8

[7] Hor CHH , Tang BL , Goh ELK . Rab23 and developmental disorders. Rev Neurosci. 2018;29:849‐860. 29727300 10.1515/revneuro-2017-0110

[8] Butler MG , Rafi SK , Manzardo AM . High‐resolution chromosome ideogram representation of currently recognized genes for autism spectrum disorders. Int J Mol Sci. 2015;16:6464‐6495. 25803107 10.3390/ijms16036464 PMC4394543

[9] Xue H , Tian GY . MiR‐429 regulates the metastasis and EMT of HCC cells through targeting RAB23. Arch Biochem Biophys. 2018;637:48‐55. 29191386 10.1016/j.abb.2017.11.011

[10] Chen Y , Ng F , Tang BL . Rab23 activities and human cancer‐emerging connections and mechanisms. Tumour Biol. 2016;37:12959‐12967. 27449041 10.1007/s13277-016-5207-7

[11] Zhang L , Zhang B , You W , Li P , Kuang Y . Rab23 promotes hepatocellular carcinoma cell migration via Rac1/TGF‐β signaling. Pathol Oncol Res. 2020;26:301‐306. 30191377 10.1007/s12253-018-0463-z

[12] Gao L , Zheng M , Guo Q , et al. Downregulation of Rab23 inhibits proliferation, invasion, and metastasis of human ovarian cancer. Int J Biochem Cell Biol. 2019;116:105617. 31550546 10.1016/j.biocel.2019.105617

[13] Liu Y , Zeng C , Bao N , et al. Effect of Rab23 on the proliferation and apoptosis in breast cancer. Oncol Rep. 2015;34:1835‐1844. 26238143 10.3892/or.2015.4152

[14] Liu YJ , Wang Q , Li W , et al. Rab23 is a potential biological target for treating hepatocellular carcinoma. World J Gastroenterol. 2007;13:1010‐1017. 17373734 10.3748/wjg.v13.i7.1010 PMC4146862

[15] Eggenschwiler JT , Espinoza E , Anderson KV . Rab23 is an essential negative regulator of the mouse sonic hedgehog signalling pathway. Nature. 2001;412:194‐198. 11449277 10.1038/35084089

[16] Sun HJ , Liu YJ , Li N , et al. Sublocalization of Rab23, a mediator of sonic hedgehog signaling pathway, in hepatocellular carcinoma cell lines. Mol Med Rep. 2012;6:1276‐1280. 23007279 10.3892/mmr.2012.1094

[17] Jeng K‐S , Chang C‐F , Lin S‐S . Sonic hedgehog signaling in organogenesis, tumors, and tumor microenvironments. Int J Mol Sci. 2020;21:758. 31979397 10.3390/ijms21030758 PMC7037908

[18] King KL , Hwang JJ , Chau GY , et al. Ki‐67 expression as a prognostic marker in patients with hepatocellular carcinoma. J Gastroenterol Hepatol. 1998;13(3):273‐279. 9570240 10.1111/j.1440-1746.1998.01555.x

[19] Craig AJ , von Felden J , Garcia‐Lezana T , Sarcognato S , Villanueva A . Tumour evolution in hepatocellular carcinoma. Nat Rev Gastroenterol Hepatol. 2020;17:139‐152. 31792430 10.1038/s41575-019-0229-4

[20] Guessous F , Li Y , Abounader R . Signaling pathways in medulloblastoma. J Cell Physiol. 2008;217:577‐583. 18651559 10.1002/jcp.21542

[21] Barginear MF , Leung M , Budman DR . The hedgehog pathway as a therapeutic target for treatment of breast cancer. Breast Cancer Res Treat. 2009;116:239‐246. 19479372 10.1007/s10549-009-0423-0

[22] Du W , Li D , Xie J , Tang P . miR‐367‐3p downregulates Rab23 expression and inhibits hedgehog signaling resulting in the inhibition of the proliferation, migration, and invasion of prostate cancer cells. Oncol Rep. 2021;46:192. 34278506 10.3892/or.2021.8143 PMC8299014

[23] Sicklick JK , Li YX , Jayaraman A , et al. Dysregulation of the hedgehog pathway in human hepatocarcinogenesis. Carcinogenesis. 2006;27:748‐757. 16339184 10.1093/carcin/bgi292

[24] Iriana S , Asha K , Repak M , Sharma‐Walia N . Hedgehog signaling: implications in cancers and viral infections. Int J Mol Sci. 2021;22:1042. 33494284 10.3390/ijms22031042 PMC7864517

[25] Ruat M , Hoch L , Faure H , Rognan D . Structure of the smoothened receptor. Med Sci. 2013;29:855‐860. 10.1051/medsci/20132910012 24148123

[26] Jeng KS , Sheen IS , Leu CM , Tseng PH , Chang CF . The role of smoothened in cancer. Int J Mol Sci. 2020;21:6863. 32962123 10.3390/ijms21186863 PMC7555769

